# Development of a high‐sensitivity ELISA detecting IgG, IgA and IgM antibodies to the SARS‐CoV‐2 spike glycoprotein in serum and saliva

**DOI:** 10.1111/imm.13349

**Published:** 2021-05-24

**Authors:** Sian E. Faustini, Sian E. Jossi, Marisol Perez‐Toledo, Adrian M. Shields, Joel D. Allen, Yasunori Watanabe, Maddy L. Newby, Alex Cook, Carrie R. Willcox, Mahboob Salim, Margaret Goodall, Jennifer L. Heaney, Edith Marcial‐Juarez, Gabriella L. Morley, Barbara Torlinska, David C. Wraith, Tonny V. Veenith, Stephen Harding, Stephen Jolles, Mark J. Ponsford, Tim Plant, Aarnoud Huissoon, Matthew K. O'Shea, Benjamin E. Willcox, Mark T. Drayson, Max Crispin, Adam F. Cunningham, Alex G. Richter

**Affiliations:** ^1^ Institute of Immunology and Immunotherapy University of Birmingham Birmingham UK; ^2^ School of Biological Sciences University of Southampton Southampton UK; ^3^ Department of Biochemistry Oxford Glycobiology Institute University of Oxford Oxford UK; ^4^ Binding Site Group Ltd Birmingham UK; ^5^ Institute of Microbiology and Infection University of Birmingham Birmingham UK; ^6^ Institute of Applied Health Research University of Birmingham Birmingham UK; ^7^ Department of Critical Care Medicine University Hospitals Birmingham NHS Trust Birmingham UK; ^8^ Immunodeficiency Centre for Wales Cardiff UK; ^9^ Department of Immunology University Hospitals Birmingham NHS Foundation Trust Birmingham UK

**Keywords:** antibodies, COVID‐19, ELISA, SARS‐CoV‐2

## Abstract

Detecting antibody responses during and after SARS‐CoV‐2 infection is essential in determining the seroepidemiology of the virus and the potential role of antibody in disease. Scalable, sensitive and specific serological assays are essential to this process. The detection of antibody in hospitalized patients with severe disease has proven relatively straightforward; detecting responses in subjects with mild disease and asymptomatic infections has proven less reliable. We hypothesized that the suboptimal sensitivity of antibody assays and the compartmentalization of the antibody response may contribute to this effect. We systematically developed an ELISA, optimizing different antigens and amplification steps, in serum and saliva from non‐hospitalized SARS‐CoV‐2‐infected subjects. Using trimeric spike glycoprotein, rather than nucleocapsid, enabled detection of responses in individuals with low antibody responses. IgG1 and IgG3 predominate to both antigens, but more anti‐spike IgG1 than IgG3 was detectable. All antigens were effective for detecting responses in hospitalized patients. Anti‐spike IgG, IgA and IgM antibody responses were readily detectable in saliva from a minority of RT‐PCR confirmed, non‐hospitalized symptomatic individuals, and these were mostly subjects who had the highest levels of anti‐spike serum antibodies. Therefore, detecting antibody responses in both saliva and serum can contribute to determining virus exposure and understanding immune responses after SARS‐CoV‐2 infection.

## Graphical abstract

This manuscript describes the development of a highly sensitive ELISA, optimizing different antigens and amplification steps, in serum and saliva from non‐hospitalized SARS‐CoV‐2‐infected subjects. Using a trimeric spike glycoprotein, rather than nucleocapsid, enabled detection of responses in individuals with mild‐to‐moderate infection. The detection of antibodies in both serum and saliva can contribute to determining virus exposure and understanding immune responses to SARS‐CoV‐2.

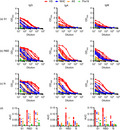

**Abbreviations**
AS: asymptomatic subject
AUC: area under the curve
BSA: bovine serum albumin
CoCo: COvid‐19 seroCOnversion study
COVID‐19: coronavirus disease 2019
DNA: deoxyribonucleic acid
ELISA: enzyme‐linked immunosorbent assay
hACE2: human angiotensin‐converting enzyme
HEK: human embryonic kidney
HRP: horseradish peroxidase
HS: hospitalized subject
N: nucleocapsid protein
NHC: non‐hospitalized convalescent
PBS: phosphate‐buffered saline
PIMS‐TS: paediatric multisystem inflammatory syndrome
RBD: receptor binding domain
RNA: ribonucleic acid
RT: room temperature
RT‐PCR: reverse transcriptase–polymerase chain reaction
S: Spike protein
SARS‐CoV‐2: severe acute respiratory syndrome coronavirus 2
SPR: surface plasmon resonance
UHB: University Hospital Birmingham

## INTRODUCTION

COVID‐19, caused by SARS‐CoV‐2, has resulted in millions of cases and more than 400 000 deaths around the world [1]. Detection of active infection is routinely achieved by testing for viral RNA, but this approach cannot be used once symptoms have resolved. Antibody testing is useful to determine historic exposure to the virus, may provide insight into the immunological status of the individual and could be a measure of protection against reinfection.

The development of novel antibody tests requires a comprehensive understanding of the humoral response to a specific pathogen across the spectrum of disease caused by that pathogen. An important factor is the variable clinical presentation of infection that can influence the concentration of antibody induced within a subject. Understanding antibody responses in individuals with the lowest symptomatology will be of major importance for monitoring viral transmission within this SARS‐CoV‐2 pandemic [2]. We have previously reported that asymptomatic seroconversion associates with lower levels of antibody to viral spike protein, which may complicate discriminating between asymptomatically infected individuals and those who were never infected [3]. Antigen choice and purity are other elements that can influence performance of the assay, not least by detecting cross‐reactive antibodies induced by previous infection to other coronaviruses.[4] Therefore, the development of assays to detect low levels of antiviral antibodies need to consider multiple variables in order to be of use in seroepidemiological studies.

Understanding the relationship between the varied clinical presentations of COVID‐19 and the serological response that arises during and following infection will be of major significance in understanding the immunopathogenesis of disease and selecting appropriate treatments. This includes the degree of antigen recognition and the antibody subclasses involved. Little is known about the role of different antibody subclasses offering protection versus driving immunopathology in COVID‐19. For instance, antibodies such as IgM, and the IgG subclasses IgG1 and IgG3 are efficient at activating complement, whereas IgA and IgG2 are not [5].

Most pathogens that enter via mucosal surfaces can induce immune responses within the mucosa and associated secondary lymphoid organs and systemic immunity in distant lymphoid organs, such as the spleen. Systemic and mucosal immune responses can share significant overlap, yet the two immune systems are semi‐autonomous. Nevertheless, other studies have shown that mucosal immunity can drive systemic responses, demonstrating that an interrelationship often occurs [6, 7]. In the context of SARS‐CoV‐2 infection, the relationship between systemic and mucosal antibody responses is not completely understood. Assessing and understanding this aspect is important as it offers the opportunity to simplify testing through use of less invasive approaches, for example using saliva. Undermining the ready use of tests that examine salivary antibody levels is that levels against specific pathogens can be a 100‐ to a 1000‐fold less than serum levels and thus fall under the level of detection of assays employed [8]. Mucosal antibody studies may also provide insights into the nature of post‐infection protective immunity and help us understand the interrelationship between systemic and mucosal immunity to the virus, which has applications for vaccine programmes.

In this study, we report on the use of an antibody assay to detect antibodies in subjects with lower levels of SARS‐CoV‐2 specific antibody. To do this, we examined responses to two well‐characterized proteins – the surface‐exposed spike (S) protein that is a target of neutralizing antibodies and the nucleocapsid (N) protein, which is the most abundant viral protein. After identifying the optimal approach to maximize the signal:noise ratio, we then determined the relationship between antibodies in serum and saliva. This work will help accelerate the development of sensitive ELISA methods available to researchers and also inform on short‐ and long‐term immunity.

## METHODS

### Patient cohorts and ethical review

Paired serum and saliva samples were collected from healthcare workers at University Hospitals Birmingham NHS Foundation Trust as part of the CoCo study. The study was approved by the London – Camden & Kings Cross Research Ethics Committee reference 20/HRA/1817. Pre‐2019 negative controls were recruited as part of a University of Birmingham study – reference ERN_16‐178. All participants in both studies provided written, informed consent prior to their enrolment. Surplus serum samples from individuals with a history of PCR‐proven SARS‐CoV‐2 infection at University Hospitals Birmingham NHS Foundation Trust and the Immunodeficiency Centre for Wales were fully anonymized and used for assay development and quality assurance. To ensure that the ELISAs could detect the range of antibodies generated by the spectrum of disease severity, we tested convalescent samples from patients hospitalized on the intensive care unit and community patients with a milder phenotype. We also tested samples previously acquired pre‐2019 before the pandemic began as these samples would be truly negative.

**FIGURE 1:**
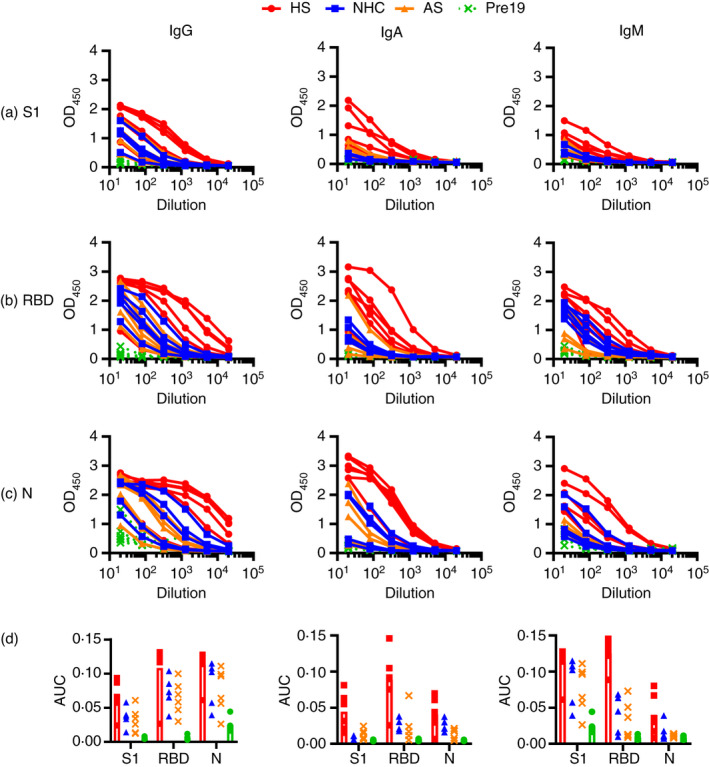
Hospitalized patients respond strongly to multiple viral proteins. Serological responses from hospitalized (HS, *n* = 6), non‐hospitalized convalescents (NHC, *n* = 5), RT‐PCR+asymptomatic subjects (AS, *n* = 6) or pre‐2019 normal donors (Pre19, *n* = 6) as determined by ELISA using HRP‐labelled anti‐IgG, IgA and IgM, against 0·1 µg purified (a) viral spike protein S1 fragment (S1), (b) receptor binding domain (RBD) or (c) nucleocapsid (N). (d) Area under the curve [11] of responses shown in a–c. The mean ± standard deviation of the mean (SD) is plotted

The serum samples from the hospitalized subjects (HS) in Figures 1 (*N* = 6), 2 (*N* = 5) and 3 (*N* = 3) were surplus samples from individuals with proven PCR‐positive infection at UHB from 04/03/2020 to 25/03/2020. The surplus serum samples from UHB were taken on during the first wave of the COVID‐19 pandemic. The asymptomatic PCR‐positive serum samples (Figure 1; *N* = 6) were from a cross‐sectional study of asymptomatic healthcare workers undertaken on the 24 and 25 April 2020 during the first wave of the COVID‐19 pandemic at the UHB NHS Foundation Trust. The non‐hospitalized convalescent (NHC) serum samples included in Figure 1 (*N* = 5) were surplus samples from healthcare workers who were proven PCR‐positive at the Immunodeficiency Centre for Wales. These samples were collected from 01/04/2020 to 01/05/2020. The NHC samples in Figure 3 (*N* = 3) were also collected at the centre above from 13/04/2020–29/04/2020. The NHC samples in Figure 4 (*N* = 20) and Figure S1 (*N* = 11) were also collected at the centre above from 29/03/2020 to 06/04/2020. The paired serum and saliva samples in Figure 5 (*N* = 80) from non‐hospitalized healthcare workers (NHC) at University Hospitals Birmingham (UHB) NHS Foundation Trust as part of the CoCo study were collected between 27/04/2020 and 08/06/2020 during the first wave of the COVID‐19 pandemic. The staff recruited had previously self‐isolated because they experienced symptoms of COVID‐19 or self‐isolated because household contacts had experienced symptoms of COVID‐19. Time from symptom onset in the above cohorts is outlined in Table S1.

**FIGURE 2:**
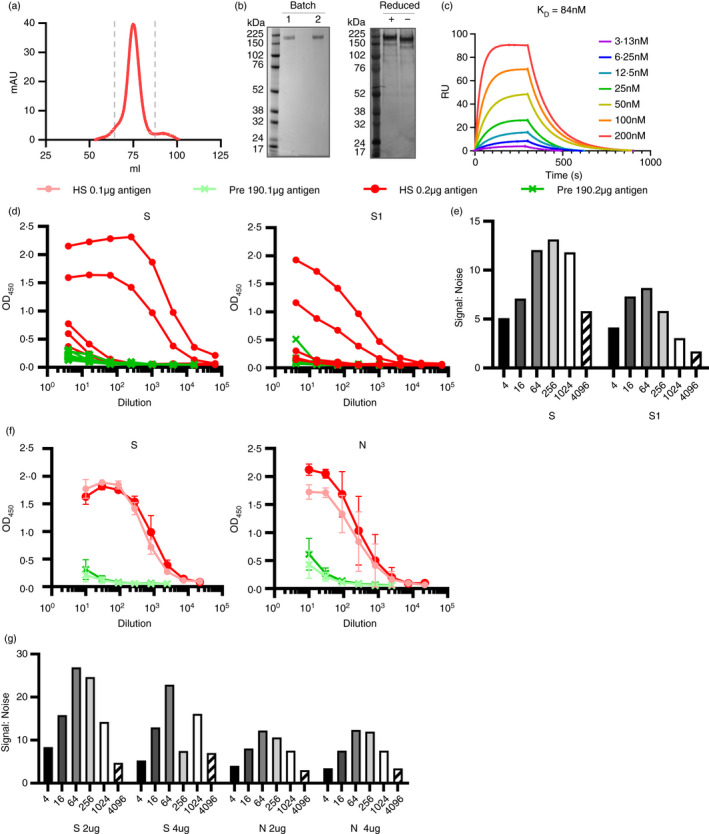
Stabilized, trimeric S antigen is a superior antigen to detect Ab in NHC. (a) Size‐exclusion chromatogram (SEC) for SARS‐CoV‐2 S protein fractions collected for further use and denoted by dashed grey lines. (b) Coomassie‐stained SDS‐PAGE gel for two separate expressions of SARS‐CoV‐2 (left) and silver stain of batch 1 under reducing and non‐reducing conditions (right). (c) Surface plasmon resonance (SPR) characterizing the interaction between SARS‐CoV‐2 S protein and Ace2. The plotted lines represent the averages of three analytical repeats at each concentration. (d) Serological responses from hospitalized (HS, *n* = 5) or pre‐2019 normal donors (Pre19, *n* = 6) as determined by ELISA using HRP‐labelled anti‐IgG represented as absorbance values or (e) signal:noise ratio at each serum dilution against 0·1 µg purified viral trimeric spike protein (S) or the S1 fragment (S1). (f) Mean absorbance values of 4 sera per group against 0·1 or 0·2 µg S or nucleocapsid (N). (g) Signal:noise ratio at each serum dilution against 0·1 or 0·2 µg of S or N. Error bars represent standard deviation from the mean (SD)

**FIGURE 3:**
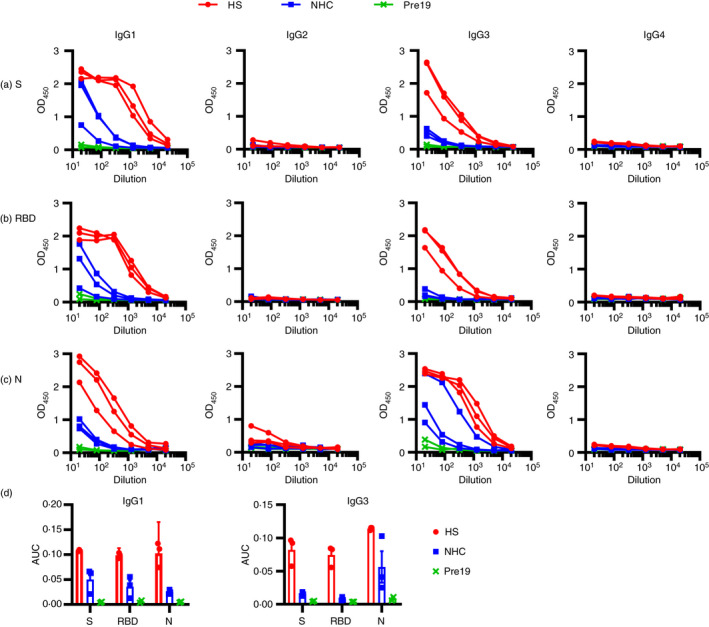
Antigen targeting and antibody isotypes do not differ depending upon the severity of disease. Serological responses from hospitalized (H, *n* = 3), non‐hospitalized convalescent (NHC, *n* = 3) or pre‐2019 donors (Pre19, *n* = 2) as determined by ELISA using HRP‐labelled anti‐IgG1, IgG2, IgG3 or IgG4 against 0·1 µg (a) trimeric spike protein (S), (b) receptor binding domain (RBD) or (c) nucleocapsid (N). (d) Area under the curve [11] of IgG1 and IgG3 responses as shown in a–c. The mean ± standard deviation of the mean (SD) is plotted

**FIGURE 4:**
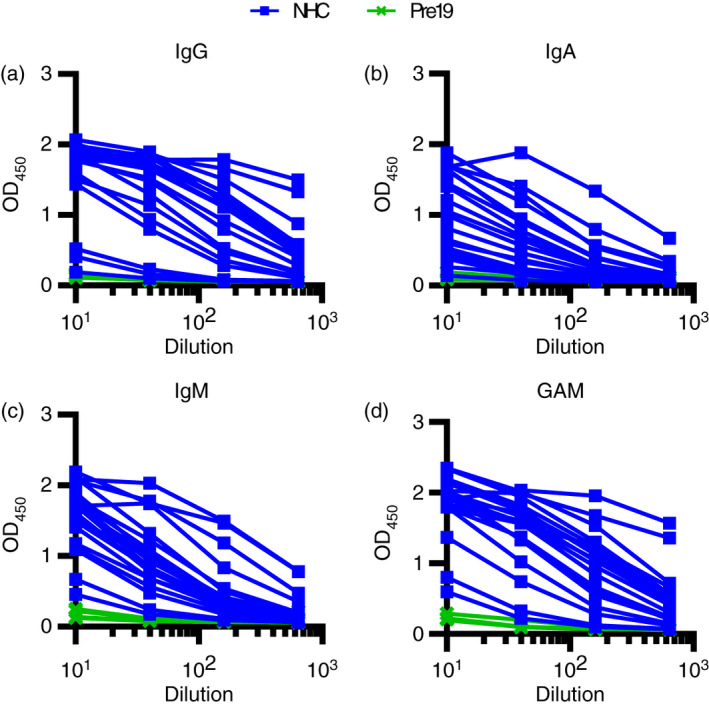
Combined detection of IgG, IgA and IgM enhances discrimination of infected and Pre19 groups. Serological responses from non‐hospitalized convalescents (NHC, *n* = 20) or pre‐2019 donors (Pre19, *n* = 4) as determined by ELISA using HRP‐labelled (a) anti‐IgG, (b) IgA and (c) IgM or (d) combined GAM, against 0·1 µg purified viral spike protein (S)

**FIGURE 5:**
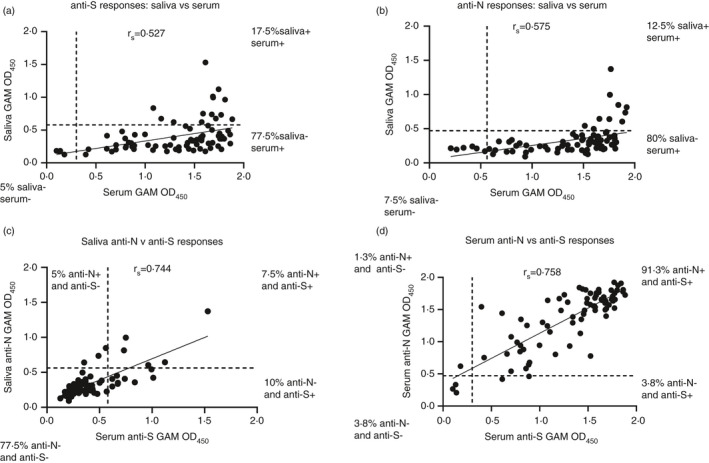
Serum and salivary anti‐SARS‐CoV‐2 antibody responses exhibit a moderate correlation. Absorbance values of paired serum diluted 1:40 and saliva diluted 1:2 from RT‐PCR‐positive convalescent healthcare workers (*n* = 80) who were symptomatic at the time of testing serum, as determined by ELISA using combined HRP‐labelled anti‐IgG, IgA and IgM [23] for serum and unlabelled combined anti‐IgG, IgA and IgM with a tertiary HRP‐labelled goat anti‐mouse Ig amplification step for saliva against 0·1 µg trimeric spike protein (S) or 0·2 µg nucleocapsid (N). (a) Correlation between paired serum and saliva absorbance values with percentages of samples positive for anti‐S antibodies in either serum, saliva or both. Positivity was determined by cut‐offs for each fluid (dotted lines) based on the mean +2 standard deviations of 8 pre‐2019 (Pre19) negative samples for sera and 83 pre‐2019 negative samples for saliva. Solid line represents simple linear regression of all samples. (b) Correlation between paired serum and saliva absorbance values with percentages of samples positive for anti‐N antibodies in either serum, saliva or both. Positivity was determined by cut‐offs for each fluid (dotted lines) based on the mean +2 standard deviations of 8 pre‐2019 (Pre19) negative samples for sera and 83 pre‐2019 negative samples for saliva. Solid line represents simple linear regression of all samples. (c) Correlation between paired saliva absorbance values with percentages of samples positive for anti‐S and anti‐N antibodies in saliva. Positivity was determined by cut‐offs for saliva (dotted lines) based on the mean +2 standard deviations of 83 pre‐2019 negative samples for saliva. Solid line represents simple linear regression of all samples. (d) Correlation between paired saliva absorbance values with percentages of samples positive for anti‐S and anti‐N antibodies in serum. Positivity was determined by cut‐offs for serum (dotted lines) based on the mean +2 standard deviations of 8 pre‐2019 negative samples for serum. Solid line represents simple linear regression of all samples

### Sample collection

Serum samples were obtained from whole blood after centrifugation at 1643*g* for 5 min and were stored at −20° until used in the assay. Whole saliva samples were collected by passive dribble into 50‐ml saliva collection tubes for a timed period of 4 min. All saliva samples were stored/transported on ice upon receipt of the laboratory for processing in order to preserve sample integrity. Samples were centrifuged (2147*g* for 10 min) to separate cells and insoluble matter, and the supernatant was removed and was frozen/stored at −20° on the same day until use. On the day of assay, samples were thawed and microcentrifuged (10621*g* for 10 min).

### Source and preparation of viral antigens

Where stated in Figures 1 and 2, the following commercial antigens were used: the S1 subunit of the S glycoprotein and nucleocapsid (N) proteins from the Native Antigen Company; SARS‐CoV‐2 spike glycoprotein (S1), product code: REC31806‐500; and His‐tagged SARS‐CoV‐2 nucleoprotein (N) from *E*. *coli*, product code: REC31851‐500.

In all other instances, antigens were prepared in house for these studies. The receptor binding domain (RBD) was generated at the University of Birmingham. Briefly, the sequence encoding RBD (amino acids 319–541) of the SARS‐CoV‐2 spike protein including a C‐terminal hexahistidine tag in the pCAGGS mammalian expression vector was obtained from Florian Krammer (Icahn School of Medicine at Mount Sinai, New York) [9]. This construct was used to transiently transfect 293 T cells cultured in Opti‐MEM (Thermo Fisher Scientific) in 2‐L roller bottles using polyethylenimine [4] linear (Polysciences, Inc, USA). The supernatant was harvested on day 4 after transfection, dialysed into PBS overnight and loaded onto a Ni‐NTA Agarose (Qiagen) column by gravity flow. The column was washed with PBS containing 10 mM imidazole, eluted using 250 mM imidazole in PBS, then buffer‐exchanged into PBS using a PD10 column (GE Healthcare).

The expression plasmid encoding SARS‐CoV‐2 S glycoprotein [10] was transiently transfected into human embryonic kidney (HEK) 293F cells. Cells were maintained at a density of 0·2–3 × 10^6^ cells per ml at 37°, 8% CO_2_ and 125 rpm shaking in FreeStyle 293F media (Fisher Scientific). Prior to transfection, two solutions containing 25 ml Opti‐MEM (Fisher Scientific) medium were prepared. Plasmid DNA was added to one to give a final concentration after transfection of 310 μg/l. Polyethylenimine [4] max reagent (1 mg/ml, pH 7) was added to the second solution to give a ratio of 3:1 PEI max: plasmid DNA. The two solutions were combined and incubated for 30 min at room temperature. Cells were transfected at a density of 1x10^6^ cells per ml and incubated for 7 days at 37° with 8% CO_2_ and 125 rpm shaking.

After harvesting, the cells were spun down at 4000 rpm for 30 min, and the supernatant was applied to a 500 ml Stericup‐HV sterile vacuum filtration system (Merck) with a pore size of 0·22 µm. The supernatant containing SARS‐CoV‐2 S protein was purified using 5 ml HisTrap FF column connected to an Akta Pure System (GE Healthcare). Prior to loading the sample, the column was washed with 10 column volumes of washing buffer (50 mM Na_2_PO_4_, 300 mM NaCl) at pH 7. The sample was loaded onto the column at a speed of 2 ml/min. The column was washed with washing buffer (10 column volumes) containing 50 mM imidazole and eluted in 3 column volumes of elution buffer (300 mM imidazole in washing buffer). The elution was concentrated by a Vivaspin column (100 kDa cut‐off) to a volume of 1 ml and buffer‐exchanged to phosphate‐buffered saline (PBS).

The Superdex 200 16 600 column was washed with PBS at a rate of 1 ml/min. After 2 h, 1 ml of the nickel affinity‐purified material was injected into the column. Fractions separated by SEC were pooled according to their corresponding peaks on the size‐exclusion chromatograms. The target fraction was concentrated in 100 kDa Vivaspin (GE healthcare) tubes to ~1 ml.

### ACE2 expression and purification

To determine the functionality of the purified SARS‐CoV‐2 S glycoprotein, SPR (surface plasmon resonance) was performed using truncated soluble angiotensin‐converting enzyme 2 (hACE2). This construct is identical to full‐length ACE2 except that it is truncated at position 626. This protein was expressed and purified identically as for the SARS‐CoV‐2 glycoprotein, with the exception of a smaller Vivaspin cut‐off being used for buffer exchanging. Following purification, the His‐tag was removed from ACE2 using HRV3C protease cleavage (Thermo Fisher). Digestion was performed at a ratio of 1:20 HRV3C protease: ACE2 in 1x HRV3C reaction buffer (Thermo Fisher), and incubated at 4° overnight. To remove the HRV3C and uncleaved ACE2, nickel affinity chromatography was performed, except that the flow through was collected rather than the elution.

### Surface plasmon resonance

After removing metallic contaminants via a pulse of EDTA (350 mM) for 1 min at a flow rate of 30 μl/min, the chip was loaded with Ni^2+^ by injecting NiCl_2_ for 1 min at a flow rate of 10 μl/min. SARS‐CoV‐2 S protein (50 mg/ml) was injected at 10 μl/min for 3 min. Control channels received neither trimer nor NiCl_2_. Control cycles were performed by flowing the analyte over Ni^2+^‐loaded NTA in the absence of trimer; there were no indications of non‐specific binding. The analyte was injected into the trimer sample and control channels at a flow rate of 50 μl/min. Serial dilutions ranging from 200 nM to 3·125 nM were performed in triplicate along with HBS P+ buffer only as a control. Association was recorded for 300 s and dissociation for 600 s. After each cycle of interaction, the NTA‐chip surface was regenerated with a pulse of EDTA (350 mM) for 1 min at a flow rate of 30 μl/min. A high flow rate of analyte solution (50 μl/min) was used to minimize mass‐transport limitation. The resulting data were fit to a 1:1 binding model using Biacore Evaluation Software (GE Healthcare), and these fitted curves were used to calculate *K*_D_.

### ELISA methodology

96‐well high‐binding plates (Corning, USA) were coated overnight at 4° with antigens at the stated dilutions in sterile PBS. Plates were blocked with 2% BSA (Sigma, UK) prepared in PBS‐0·1% Tween‐20 for 1 h at room temperature (RT). Pre‐diluted serum or saliva samples were added (100 µL per dilution) and serially diluted and plates incubated for 1 h at RT. After washing with PBS‐0·1% Tween, 100 µl of HRP‐conjugated or unconjugated mouse anti‐human immunoglobulins were added and incubated for 1 h at RT. Anti‐human immunoglobulin antibodies (anti‐IgG, clone R‐10 1:8000; anti‐IgA clones, 2D7 (which binds to IgA1 and IgA2) 1:2000 and MG4.156 1:4000; anti‐IgM clone AF6 1:2000; anti‐IgG1 clone MG6.41, 1:3000; anti‐IgG2 clone MG18.02 1: 3000; anti‐IgG3 clone MG5.161 1:1000; anti‐IgG4 clone RJ4 1:1000) are all clones generated in the University of Birmingham and available from Abingdon Health, UK [11, 12, 13, 14]. In some experiments, HRP‐labelled goat anti‐mouse immunoglobulins (Southern Biotech, USA) were added and incubated for 1 h at RT. After washing, plates were developed for 5–10 min with 100 µl of TMB core (Bio‐Rad, UK) and then stopped with 50 µl of 0·2 M H_2_SO_4_. OD was recorded at 450 nm using the Dynex DSX automated liquid handler (Dynex Technologies, USA). Signal:noise ratio (S:N ratio) was calculated by dividing the average OD from the positive samples (signal) over the average OD from the pre‐2019 negative controls (noise).

### Statistical analysis

Data were checked for normality using the Wilk–Shapiro test. Pairwise Spearman's rank–order correlation coefficients were used to assess the correlation of matched serum and saliva data. The significance level was assumed a priori to be 0·05. The analysis was conducted using STATA 16.1 (StataCorp LLC, USA).

## RESULTS

### Hospitalized patients induce robust responses to multiple SARS‐CoV‐2 antigens

To identify the antibody response to the virus, we tested sera against a range of viral antigens. There were three groups of subjects analysed: hospitalized subjects (HS, *N* = 14), which included individuals who were admitted to the hospital and had confirmed SARS‐CoV‐2 infection by PCR; non‐hospitalized convalescent (NHC, *N* = 39) subjects, who were patients with confirmed SARS‐CoV‐2 infection by PCR but were not hospitalized; and asymptomatic non‐hospitalized convalescent patients (AS, *N* = 6), who were individuals without reported symptoms who gave a positive result for SARS‐CoV‐2 infection by PCR. As a negative control group, we used sera taken before 2019 (Pre19, *N* = 22). Further details can be found in Table S1. Initial studies focused on the two major targets of antibody responses: the viral S1 fragment and purified receptor binding domain (RBD) of spike (S) glycoprotein and nucleocapsid (N) proteins. As expected, strong IgG, IgA and IgM responses were detected to these proteins in all HS individuals with severe disease (Figure 1). In contrast to the strong responses observed in severe cases, lower IgG, IgA and IgM responses were observed in the NHC subjects, and in some instances, responses were undetectable (Figure 1b and Figure S1). Area under the curve [11] calculations confirmed that the highest response was observed in the group of HS for all the antigens tested (Figure 1d). However, in RT‐PCR‐confirmed AS and NHC subjects, responses were more similar to one another and typically lower than in HS for all isotypes (Figure 1d). Non‐parametric 2‐tailed Mann–Whitney *U* *t*‐tests were performed to detect differences between the HS and NHC cohorts, and there were no differences found (*P* = 0·208 (NS)). Thus, simple non‐optimized ELISAs readily detect antibodies to spike, RBD and N protein in sera from RT‐PCR‐confirmed COVID‐19 patients.

### Generation of soluble, native‐like trimeric S glycoprotein

The use of RBD and S1 fragments within the assay was sufficient to detect antibodies in most individuals. Nevertheless, we hypothesized that these subunits may result in suboptimal detection of antibodies in sera, particularly where titres were low, as these constructs both present intrinsically lower number of native epitopes and, in the case of the RBD, additionally display non‐native epitopes hidden in the natively folded glycoprotein. Therefore, we produced soluble trimeric SARS‐CoV‐2 S glycoprotein. We expressed and purified recombinant SARS‐CoV‐2 S glycoprotein containing the previously described 2P‐stabilized form, with a construct lacking the furin cleavage site, which minimizes S1/S2 subunit shedding, and is trapped in the pre‐fusion conformation [10]. The purity of the resultant SARS‐CoV‐2 S glycoprotein was confirmed by both SDS‐PAGE (Figure 2a,b) and mass spectrometry (reported in detail elsewhere [15]). To ensure the SARS‐CoV‐2 was natively folded, we performed surface plasmon resonance (SPR) between the purified S glycoprotein and its cognate receptor human angiotensin‐converting enzyme (hACE2) (Figure 2c). We determined a *K*_D_ of 84 nM for hACE2 binding to SARS‐CoV‐2 S glycoprotein confirming the functionality of the purified glycoprotein.

### Native‐like, trimeric S antigen is superior to N to detect AB in sera at higher dilutions

We then assessed whether changing multiple parameters within the assay could enhance the responses detected. By using the purified trimeric S glycoprotein, we enhanced antibody detection compared with S1 protein, both in terms of the absolute OD_450_ values and in terms of the signal:noise ratio, and this was particularly notable as antibody became limiting (Figure 2d,e). For example, the signal:noise ratio when S glycoprotein was used was above 10 and only dropped when the sera was diluted to 1:4096. In contrast, signal:noise ratio for S1 remained lower than 10 at all dilutions tested. Increasing the amount of S glycoprotein per well from 0·1 to 0·2 µg/well modestly enhanced the anti‐S OD_450_ values (Figure 2f), but overall provided no improvement of the signal:noise ratio (Figure 2g). Similarly, higher concentrations of N did improve signal detection but had little difference to the signal:noise ratio, as background responses to control sera also increased (Figure 2f,g). Thus, total trimeric S glycoprotein provides better discrimination to identify infected from non‐infected individuals than S1 or N protein.

### Antibody subclass distribution does not differ depending upon the severity of disease

We then investigated the type of antibody response in patients by identifying the IgG subclasses generated against each antigen, and whether these responses varied to the different antigens. This matters beyond the seroepidemiological detection of infection because heavy chain use influences the effector function of antibodies. In HS and NHC, IgG1 and IgG3 were detected to S, RBD and N (Figure 3a–c). However, AUC for IgG1 and IgG3 was lower in NHC compared with HS (Figure 3d). IgG2 was largely undetectable regardless of the antigen or the origin of the sera. However, some samples were weakly positive for IgG4 to N but not S (Figure 3c). The IgG1 response predominated against S or its RBD component. Thus, severe and less severe SARS‐CoV‐2 infections result in similar IgG antibody isotype switching profiles, although the extent of IgG1 and IgG3 isotype switching may differ between antigens. Non‐parametric 2‐tailed Mann–Whitney *U* *t*‐tests were performed to detect differences between the HS and NHC cohorts, and there were no differences found (*P* > 0·999 (NS)).

### Combined detection of IgG, IgA and IgM enhances detection of antibody responses

The trimeric S glycoprotein was then used in the immunoassay to detect IgG, IgA and IgM in sera from NHC (Figure 4a–c). All antibody isotypes were detectable in the same individuals (Figure 4). As a significant need in a test is to discriminate between infected individuals with low levels of antibody and non‐infected individuals, we assessed whether combining anti‐IgG, IgA and IgM (anti‐GAM) secondary antibodies to detect all three isotypes could enhance signal detection. Merging secondary antibodies to detect anti‐GAM responses provided a signal at each dilution that reflected the strongest signal from each of the three individual isotypes (Figure 4d).

We examined whether the IgG, IgA, IgM and GAM signals to S glycoprotein could be enhanced in a subset of sera from NHC subjects who had lower levels of antibody. To do this, we included an additional tertiary amplification step whereby after labelling primary antibodies with unconjugated mouse anti‐human antibodies, HRP‐conjugated anti‐mouse immunoglobulin antibody was added. The inclusion of the amplification step enhanced the signal detected in nearly all samples but had little effect on the signal:noise ratio for IgG (Figure S1A,B). The enhanced signal detected for IgM and IgA (Figure S1A,B) resulted in higher signal:noise ratios for IgA and IgM, particularly at higher dilutions (S:N ratio at 1:540 dilution on pre‐amplification 1·4 vs 2·2 post‐amplification for IgA, and 1·5 vs 2·1 for IgM; Figure S1B). Thus, this additional step could be utilized to enhance signal detection when specific anti‐S antibodies are present at lower concentrations.

### Anti‐S or anti‐N IgGAM antibodies are not detectable in saliva from most NHC subjects

Saliva is an easily accessible fluid that can be self‐collected, is non‐invasive and could be beneficial for mass‐scale seroprevalence studies. Moreover, entry of the virus is via the upper respiratory tract and antibodies in saliva may provide a first barrier to entry at this point. Therefore, we used the ELISA, with an amplification step, to investigate anti‐S and anti‐N antibody responses in saliva from 80 NHC subjects with RT‐PCR‐confirmed infections who were recruited from the University Hospitals Birmingham NHS Foundation Trust. In parallel, we examined serum responses from these same individuals using the non‐amplified ELISA. The matched saliva and sera were tested at a 1:2 dilution and 1:40 dilution, respectively.

To determine positivity, a cut‐off value for saliva was generated by testing 83 Pre19 saliva samples (Figure S2B). The cut‐off was taken as the mean +2 SD of this population. An equivalent cut‐off was generated using 8 Pre19 sera (Figure S2A). When the NHC samples were assessed for anti‐ S glycoprotein IgGAM antibodies (Figure 5a), then the majority of saliva were negative (82·5%). Most individuals were positive only in serum (77·5%), with only 17·5% of individuals positive in both serum and saliva. Individuals who had positive results for both serum and saliva tended to have the highest OD_450_ values for serum. There was a moderate correlation between the samples (*r*_s _= 0·527, *P* < 0·001). A similar distribution of responses was observed for antibody responses to N protein with 80% of individuals only having detectable antibodies to N protein in serum; the correlation of saliva and serum samples was of moderate strength (*r*_s _= 0·575, *P* < 0·001, Figure 5b). A lower proportion of individuals were positive in both saliva and serum (12·5%): again, positive responses in saliva were mainly detected in those with the highest responses in serum. A higher proportion of individuals (7·5%) had negative responses to N protein in both serum and saliva than for S glycoprotein. Therefore, antibody responses to S and N proteins in saliva were not detectable in most RT‐PCR‐confirmed non‐hospitalized convalescents. Plotting the OD_450_ values for S and N from saliva or serum showed that there was a strong, positive correlation between responses (*r*_s _= 0·744, *P* < 0·001 and *r*_s _= 0·758, *P* < 0·001, respectively; Figure 5c,d), suggesting that individuals who made responses to one antigen made responses to the other.

## DISCUSSION

The need to be able to identify those who have previously had a SARS‐CoV‐2 infection has resulted in the development of immunoassays that are designed to measure antibodies as a signature of exposure, and there is a need to make these as sensitive and specific as possible. Antibody assays have also shown potential in diagnosing SARS‐CoV‐2‐associated complications, such as helping diagnose children who present with PIMS‐TS (paediatric multisystem inflammatory syndrome), yet are RT‐PCR‐negative for virus, and to define seroprevalence in symptomatic and asymptomatic healthcare workers in a hospital setting [3, 16, 17]. These real‐world examples of the use of this assay emphasize the potential for these studies to aid in diagnosis and immunosurveillance. If post‐infection complications arise from COVID‐19, then the availability of high‐quality assays to detect prior infection will be of obvious benefit. Collectively, these points add to the wealth of evidence supporting the benefit and value of antibody assays in the current crisis.

Our method focused on implementing different approaches to find the best signal:noise ratio. To do this, we used sera obtained from subjects prior to 2019 and sera from patients with confirmed RT‐PCR infection with different severities of disease. Within these sera from infected patients, we were particularly interested in enhancing the signal:noise ratio in sera that either contained lower levels of antibody or the ratio when sera were diluted. RBD, S1 and N were excellent at detecting antibodies in sera from subjects with severe COVID‐19, but were not as good as purified, whole, trimeric S when antibody levels were more limiting. This is unlikely to be due to the source of antigen preparations as we compared commercially purchased N, as well as antigens made within our own facility. Wider use of S glycoprotein in assays has been facilitated by recent improvements that have increased yields as much as 10‐fold [18]. Due to higher protein yields obtained after purification from culture, some studies suggest the use of RBD as a first screening test [19]. Our data support this, demonstrating higher signal:noise ratios for RBD compared with S1 in an unoptimized system. This is in contrast to Amanat et al (2020), who found S1 has higher AUCs than RBD [20]. These differences are most likely accounted for by subtle differences in the design of the ELISA used in each case. However, both RBD and S1 have a more limited set of native epitopes in comparison with the whole native S glycoprotein and, in our study, this potentially affected the signal:noise ratio, particularly when antibody concentrations are limiting.

It could be hypothesized that a greater diversity of epitopes is beneficial as there is more opportunity to capture a wider range of antibody specificities, and this may be more important when antibodies are rarer in a patient sample. Using the trimeric S glycoprotein may also help detect antibodies that block both binding and viral entry, which may offer additional insights than examining responses to RBD alone. Coupled with this is the presence of non‐native epitopes in the RBD that are not present in the whole native trimeric S glycoprotein, and the purity of the S glycoprotein generated, with the glycan profiles offering an additional quality assurance step in determining purity.

In our modified ELISA method, S protein gave a better discrimination between SARS‐CoV‐2‐positive and SARS‐CoV‐2‐negative samples than N. One concern of using the extracellular region of S in assays has been the risk of detecting antibodies that are cross‐reactive between SARS‐CoV‐2 and other coronaviruses. The level of amino acid conservation of S with other human pathogen members of the coronavirus family, such as HCoV‐OC43 and HCoV‐229E, is lower than SARS‐CoV. HCoV‐OC43 and HCoV‐229E account for 5%–30% of upper respiratory tract infections, and most individuals who are infected with these strains do so from an early age [21, 22]. Additionally, the low identity of N and S from SARS‐CoV‐2 with these viruses means cross‐reactivity of antibodies to these proteins is minimal [4]. Ng et al. (2020) recently reported that a small proportion of SARS‐CoV‐2‐uninfected individuals showed evidence of IgG antibodies that could cross‐react with the S2 subunit of the SARS‐CoV‐2 S protein. It is entirely plausible that this cross‐reactivity may contribute to an enhanced IgG antibody signal although this study found it was more prevalent in adolescents compared with adults [23]. Other groups have found no evidence of cross‐reactivity when pooled human immunoglobulin products collected prior to the COVID pandemic were used in SARS‐CoV‐2 ELISAs, which should contain antibodies against endemic coronaviruses [20, 24]. As these experiments were performed, during the first wave of the COVID pandemic, a further 624 pre‐pandemic (pre‐2019) controls have been assayed on the final build of the trimeric spike ELISA as part of a validation protocol. As part of the validation protocol, ROC curves were performed with a sensitivity of 98·4% (95% CI 91·4%–99·7%) and specificity of 97·6% (95% CI 96·1%–98·5%). Seasonal coronaviruses were also investigated, and no cross‐reactivity with endemic coronaviruses or other human viruses was observed.

Studies in cohorts of SARS‐CoV‐2‐infected patients show that antibody responses develop at different rates depending on the severity of the disease. In general, antibody titres are higher in patients with critical or severe disease when compared to those with milder disease [25, 26]. Likewise, in our investigations, we found that samples from hospitalized patients had stronger IgG, IgA and IgM responses against S1, RBD and N antigens. The important finding from these responses is that it is relatively simple to detect antibodies in patients with severe disease and that focusing on detecting responses in those who have much less severe disease or are asymptomatic may be advantageous for maximizing the sensitivity of an antibody test. Nevertheless, over time the balance between different antibody isotypes that target the spike glycoprotein can change and so the contribution of the overall signal detected may reflect the isotype balance within an individual serum. Our sample numbers were low as we primarily wanted to focus on PCR‐positive individuals, and obtaining PCR‐positive serum in the first wave of the pandemic was extremely difficult as PCR testing was not that widespread during the concept of the study and experiments that were included in this manuscript. The development of the ELISA system was then verified with much larger numbers in our validation paper [27].

When the IgG subclasses were evaluated, IgG1 and IgG3 were the most abundant in all samples tested, and they were also higher in hospitalized patients than in those with mild disease. This is important because it has been suggested that antibodies may play a role in pathogenesis, including the possible role of IgG1 as a mediator of acute lung injury in COVID‐19 [28]. Of interest, the IgG1 signal to S was consistently stronger than that of IgG3, whereas the IgG1 signal to N, a predominance of IgG1 or IgG3, was less clear. It would be a valuable study to examine IgG responses longitudinally in patients with different presentations of SARS‐CoV‐2 and examine how this relates to different presentations of infection.

Self‐collected saliva is an attractive way to evaluate antibody prevalence due to the accessibility of the sample and non‐invasiveness of the procedure, but fewer studies have explored this as a route to detect infected individuals. One reason for this is that it can be more challenging to detect antibodies in saliva. The use of an additional tertiary antibody incubation with an HRP‐conjugated goat anti‐mouse Ig to amplify the signal of the bound IgG, IgA or IgM resulted in an enhanced detection of anti‐S responses in saliva. In the matched saliva and serum samples from a cohort of RT‐PCR+‐confirmed subjects, 95% had serum IgGAM to S. Nevertheless, only 17·5% had antibodies to S in both serum and saliva and antibodies to S in saliva were mainly detected in those who had higher serum responses. This may reflect partitioning of antibody responses between mucosal and systemic sites such that only those with higher systemic responses generate responses in the saliva, or that there is less separation between mucosal and systemic responses and that serum antibodies transfer into the saliva. Similarly, but to a lesser degree, anti‐N antibodies also showed this pattern. The implications of this are unclear. It may mean this assay is suboptimal for detecting saliva antibodies. Otherwise, it may mean that many subjects do not secrete antibodies into the buccal cavity or other reasons, as yet unknown, may contribute. The nature of the cohort in these studies may also influence the proportion of subjects with saliva antibodies. The cohort contained subjects recovering from infection, and all had been symptomatic, but had not received hospital treatment. If so, it could be that the kinetics of responses are different in this NHC population compared with other's findings on salivary SARS‐CoV‐2‐specific antibodies in HS subjects. Variations in saliva sampling procedures may affect the concentration of antibody and the success in collecting mucosal antibody. There is no gold standard, and mucosal COVID antibody studies have used this technique (Isho et al.) and a gingival crevicular technique (Pisanic et al.) [29, 30]. How closely this antibody pattern in saliva represents the antibody profile from other respiratory secretions remains to be determined, but suggests that there needs to be some caution in using saliva as a testing medium to understand virus exposure. Similarly, whether the low levels of responses saliva has implications for protection is unknown as arguments could be formulated that salivary antibodies contribute to protection through providing a barrier function, or to pathology through promoting antibody‐dependent enhancements or other effects.

Therefore, standard ELISA methods based on high‐quality S protein can be modified to readily detect antibody responses in serum and saliva from individuals with SARS‐CoV‐2 infections. This method may serve as an important tool for assessing both short‐ and long‐term humoral immunity for community‐acquired COVID‐19 infections and understanding the nature of natural and vaccine‐induced protective responses to SARS‐CoV‐2 infection.

## CONFLICT OF INTEREST

AC and SH are employed by the Binding Site Group Ltd. AH has a commercial relationship with Binding Site Group Ltd. MTD and MG have a commercial relationship with Abingdon Health. The rest of the authors declared no conflict of interest.

## AUTHOR CONTRIBUTIONS

SEF, SEJ and MPT designed and performed experiments, interpreted results and wrote the manuscript. AMS interpreted results and wrote the manuscript. JDA, YW and MLN designed and performed experiments. AC performed experiments. CRW, MS and MG provided essential reagents. JLH, EMJ and GLM performed experiments. BT interpreted results. DCW interpreted results and wrote the manuscript. TVV provided essential clinical material. SH provided essential reagents. SJ and MJP provided essential clinical material. TP provided experimental support. AH provided essential clinical material. MKO interpreted results. BJE designed experiments and provided essential reagents. MTD designed experiments, interpreted results and wrote the manuscript. MC, AFC and AGR designed experiments, interpreted results, wrote the manuscript and supervised the project. All authors commented on drafts of the manuscript and approved the final version.

## Supporting information

Figure S1

Figure S2

Table S1
