# Supplementary figures and images for: Development of a high‐sensitivity ELISA detecting IgG, IgA and IgM antibodies to the SARS‐CoV‐2 spike glycoprotein in serum and saliva
Source: Immunology. 2021 May 24;164(1):135–47. doi: 10.1111/imm.13349 (PMC8242512; doi:10.1111/imm.13349)

## Slide 1
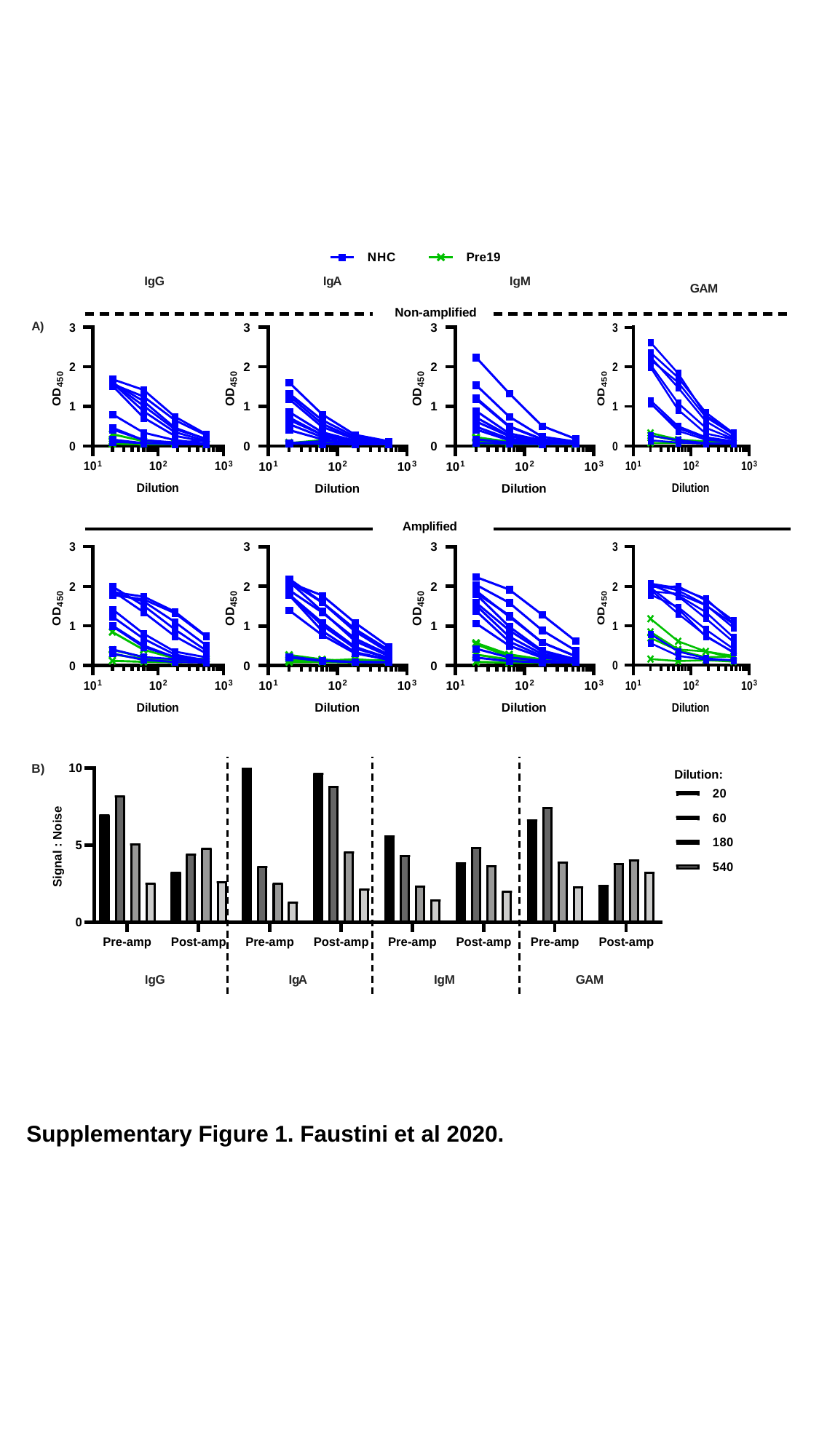

Supplementary Figure 1. Faustini et al 2020.

Supplement: Supplementary file 1 — Figure S1 [file IMM-164-135-s003.pptx]

## Slide 1
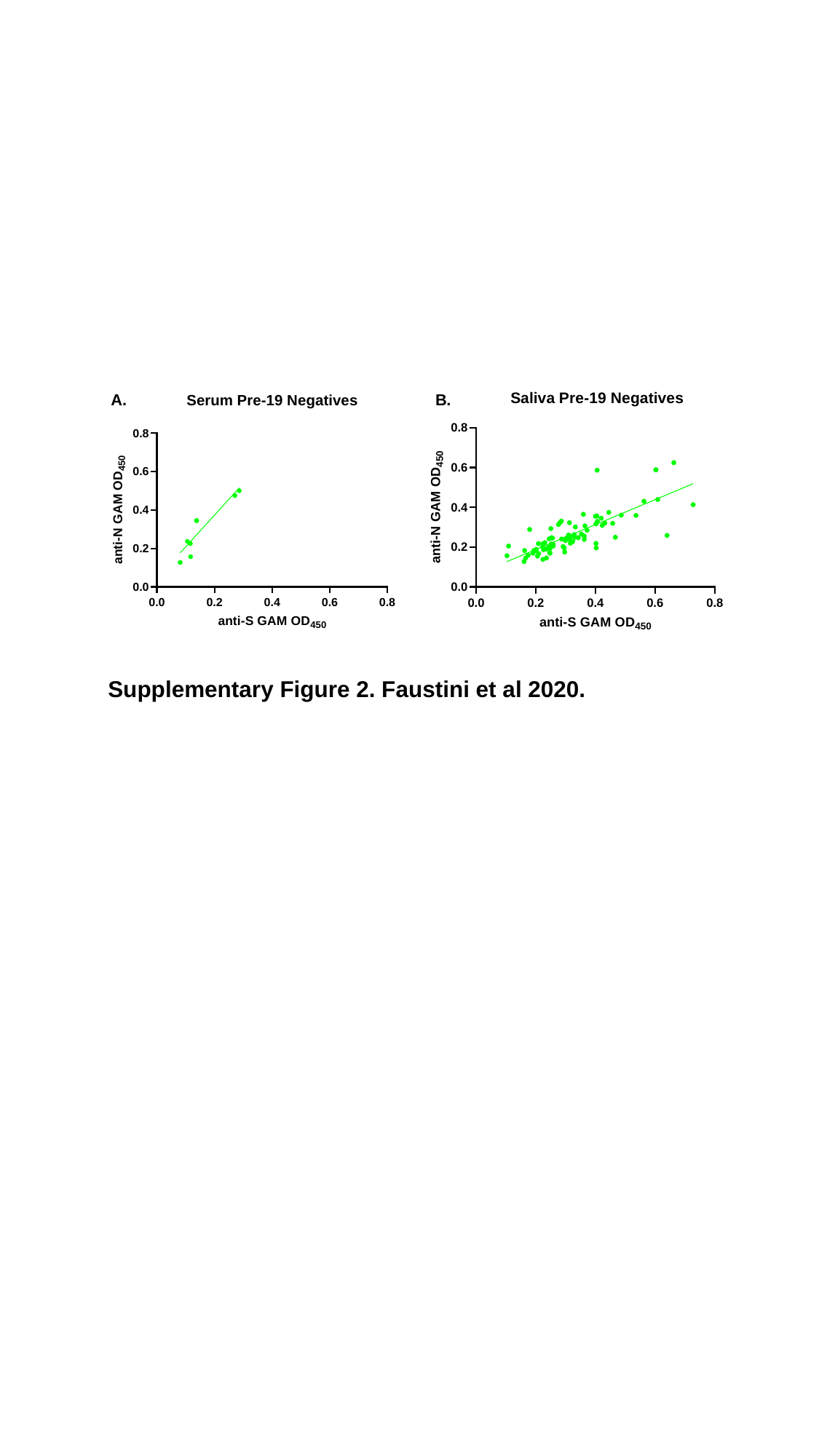

Supplementary Figure 2. Faustini et al 2020.

Supplement: Supplementary file 2 — Figure S2 [file IMM-164-135-s001.pptx]
