# Supplementary material for: Development of a high‐sensitivity ELISA detecting IgG, IgA and IgM antibodies to the SARS‐CoV‐2 spike glycoprotein in serum and saliva
Source: Immunology. 2021 May 24;164(1):135–47. doi: 10.1111/imm.13349 (PMC8242512; doi:10.1111/imm.13349)
Supplement: Supplementary file 3 — Table S1 [file IMM-164-135-s002.docx]

Supplementary Table I. Characteristics of samples tested

| ***Figure*** | ***Sample Type and Number*** | ***PCR status*** | ***Time from Symptoms Onset Median (Min-Max), days (d)*** |
| --- | --- | --- | --- |
| ***1*** | HS: N=6 | Positive | 14 (7-28) |
|  | NHC: N=5 | Positive | 23 (13-27) |
|  | AS: N=6 | Positive | N/A |
|  | Pre19: N=6 | N/A | N/A |
| ***2*** | HS: N=5 | Positive | 14 (5-32) |
|  | Pre19: N=6 | N/A | N/A |
| ***3*** | HS: N=3 | Positive | 21 (16-24) |
|  | NHC: N=3 | Positive | 17 (14-28) |
|  | Pre19: N=2 | N/A | N/A |
| ***4*** | NHC: N=20 | Positive | 14 (7-25) |
|  | Pre19: N=4 | N/A | N/A |
| ***5*** | UHB HCW/NHC serum and saliva: N=80 | Positive | 35 (13-71) |
| ***Sup. 1*** | NHC: N=11 | 10 Positive; 1 no swab available-symptomatic case exposure | 14 (7-25) |
|  | Pre19: N=4 | N/A | N/A |
| ***Sup. 2*** | Pre19 serum: N=8  Pre19 saliva: N=83 | N/A | N/A |

HS: Hospitalized subjects, NHC: Non-hospitalized Convalescent, AS: Asymptomatic Subject, UHB HCW: University Hospital Birmingham Healthcare Worker Study Subject; pre19: Pre-2019 subjects N/A: Not applied
